# Insecticides chlorantraniliprole and flubendiamide in *Aster scaber*: Dissipation kinetics, processing effects, and risk assessment

**DOI:** 10.1016/j.heliyon.2024.e33216

**Published:** 2024-06-17

**Authors:** Seung-Hyun Yang, Hoon Choi

**Affiliations:** a Department of Life and Environmental Sciences, Wonkwang University, Iksan, 54538, Republic of Korea; b Healthcare Advanced Chemical Research Institute, Environmental Toxicology and Chemistry Center, Hwasun, 58141, Republic of Korea

**Keywords:** Diamide insecticide, *Aster scaber*, Residue, Half-life, Processing

## Abstract

The residue characteristics, processing effects of washing and drying, and dietary risks of chlorantraniliprole (CAP) and flubendiamide (FBD) to Koreans were investigated using *Aster scaber* in a greenhouse. Following foliar application, the initial FBD residues were 3–10 times higher than those of CAP. However, the biological half-lives were similar at 6.0–8.3 and 6.8–9.9 days for CAP and FBD, respectively. The pre-harvest residue limits (PHRLs) 7 days before harvest, derived from the dissipation rates and maximum residue limits, were 12.2 and 33.2 mg/kg for CAP and FBD, respectively. For the removal of CAP and FBD from *A. scaber*, washing with a neutral detergent was more effective than running under or dipping in tap water (86.5 % and 66.2 %, respectively). Processing factors in fields I and II were 2.6 and 5.1 for CAP and 2.0 and 5.7 for FBD, respectively. Drying removal efficiencies in fields I and II averaged 46.4 % and 52.3 % for CAP and 48.4 % and 49.2 % for FBD, respectively. Chronic health risk assessments indicated that dietary exposure to CAP and FBD is acceptable for Korean health.

## Highlights

• Biological half-lives of CAP and FBD were 6.0–8.3 and 6.8–9.9 days in *Aster scaber*.
• Pre-harvest residue limits for *A. scaber* were determined based on dissipation rates.
• Neutral detergent washing was most effective for residue removal in *A. scaber*.
• Processing factors in the drying process were 2.6–5.1 and 2.0–5.7 for CAP and FBD.
• Chronic health risk to CAP and FBD were acceptable to the health of Koreans.

## Chemical compounds investigated in this article

Chlorantraniliprole (PubChem CID: 11271640); Flubendiamide (PubChem CID: 11193251).

## 1 Introduction

Vegetables play a vital role in promoting a healthy and balanced diet because they offer a diverse array of essential nutrients necessary for maintaining overall well-being. Recently, vegetable consumption worldwide has increased, driven by factors such as growing awareness of the health benefits of vegetables and the rising demand for fresh and nutritious food sources [1]. *Aster scaber* Thunb., commonly known as *chwinamul* in Korea, is a leafy vegetable native to East Asia, including Korea, China, and Japan. This vegetable is nutritionally rich and contains a substantial number of vitamins, minerals, essential amino acids, unsaturated fatty acids, such as linolenic acid, and dietary fiber, all of which fortify blood vessels, lower blood cholesterol, and aid in the excretion of harmful substances [2]. However, as a leafy vegetable, it is susceptible to a wide range of insect pests and diseases, resulting in a decreased yield and compromised quality. Therefore, pesticides are used during cultivation to protect crops from various insect pests and plant diseases [3].

In Korea, 61 pesticides and 376 products are registered for use in *A. scaber*. Chlorantraniliprole (CAP) and flubendiamide (FBD), which belong to the diamide insecticide class, are commonly used in Korea in addition to abamectin, chlorfenapyr, emamectin benzoate, indoxacarb, and lufenuron to control various insects, such as *Spodoptera litura*, *Spodoptera exigua*, and *Liriomyza trifolii*, which are frequent pests of *A. scaber* [4]. CAP and FBD selectively activate the ryanodine receptor in the endoplasmic reticulum and disrupt calcium ion regulation, resulting in uncontrolled muscle contractions, paralysis, and ultimately, insect death [5]. Typically, when pesticides are applied to crops, they decompose and are eliminated from plants. However, studies on plant metabolism have shown that the residues of CAP and FBD in crops following foliar application primarily comprise their parent compounds [6,7]. Consequently, for compliance with maximum residue limits (MRLs) and the estimation of dietary intake, the residues in the plant commodities have been defined for CAP and FBD. The MRLs for CAP and FBD for *A. scaber* in Korea are 7 and 20 mg/kg, respectively. For leafy vegetables, the Codex Alimentarius Commission, European Union, and United States have set MRLs for CAP and FBD at 20 and 7, 20 and 0.01, and 13 and 11 mg/kg, respectively.

Studies have reported the dissipation and dietary risk assessment of CAP and FBD in various plant samples, including brinjal, kale, paprika, perilla leaves, strawberries, tea leaves, and tomatoes [[8], [9], [10], [11], [12], [13]]. Pesticide residue levels can vary significantly depending on whether the crops are grown in a greenhouse or in open fields. Generally, greenhouse-grown crops tend to contain higher residue levels compared to those grown in open fields, as they are less exposed to factors such as wind, rainfall, and solar radiation [14,15]. The persistence of CAP and FBD in crops varies, with shorter half-lives observed in fruit crops compared to that in leafy vegetables. The half-lives of CAP can range from 10 to 19 days in perilla leaves cultivated in a greenhouse, while in strawberries and tomatoes, they can range from 3 to 6 days. Spinach, a rapidly growing crop, exhibits a half-life of 3–4 days, similar to those of fruit vegetables. However, FBD has shorter half-lives, with half-lives of 3–4 days for kale and tomatoes.

Consumers frequently engage in various processing methods such as washing, rinsing, peeling, drying, baking, extruding, and cooking before consuming fresh vegetable and fruits [[16], [17], [18]]. The efficacy of these processing techniques in the reduction of residual pesticides may vary depending on factors such as pesticide and crop types and processing conditions, including agents, duration, frequency, and temperature [17,18]. Several studies have investigated the efficacy of different processing techniques, yielding valuable insights into their effectiveness. Cui et al. [16] washed and parboiled cucumber and cowpea, successfully removing >15 % of fluxapyroxad. Jankowska et al. [17] reported that the use of water and thermal technology reduced the concentration of 21 pesticides by 6–91 % and 43–98 %, respectively, in broccoli, tomatoes, strawberries, and black currants. Yang et al. [19] demonstrated that washing with running water (77.0 ± 18.0 %) was more effective than washing with a detergent (43.7 ± 14.5 %) in the removal of ten pesticides from five types of leafy vegetables. Hwang et al. [20] showed etofenprox residue removals of 21.6–43.9 % and 66.6–88.5 % in spring onion using various washing or drying processes, respectively. Further research is needed to better understand the optimal processing methods for minimizing pesticide exposure through food consumption.

To the best of our knowledge, no previous studies have investigated the dissipation patterns of CAP and FBD in *A. scaber*. Therefore, this study aimed to investigate the residue characteristics of CAP and FBD in *A. scaber* and establish pre-harvest residue limits (PHRLs) based on their biological half-lives and dissipation constants. Additionally, this study explored the efficiency of pesticide residue removal using common processing methods, such as washing and drying, as a strategy to reduce dietary exposure to pesticides.

## 2 Materials and methods

### 2.1 Reagents and materials

The analytical standards for CAP (purity: 99.2 %) and FBD (purity: 98.3 %) were purchased from Sigma-Aldrich (St. Louis, MO, USA). Water-dispersible granules (WG, 5 %) of CAP (Altacore; Dongbu Farm Hannong, Gumi, Republic of Korea) and a suspension concentrate (SC, 20 %) of FBD (Anichung; Hankooksamgong Co., Iksan, Republic of Korea) were purchased from a commercial pesticide vendor. The variety of *A. scaber* was purchased from Asia Seed Co., Ltd. (Seoul, Republic of Korea). High-performance liquid-chromatography (HPLC)-grade acetone (ACE), dichloromethane (DCM), ethyl acetate (ETAC), and *n*-hexane (HX) were obtained from Daejung Chemicals and Metals (Siheung, Republic of Korea). HPLC-grade acetonitrile (ACN) was purchased from J. T. Baker (Avantor, Radnor, PA, USA). Deionized water (18.2 MΩ⋅cm) was prepared using a Milli-Q water purification system (Millipore, Bedford, MA, USA). Sodium chloride and anhydrous sodium sulfate were obtained from Junsei Chemical Co., Ltd. (Tokyo, Japan). Florisil (0.150–0.250 mm) and silica solid-phase extraction (SPE) cartridges (1 g, 6 cc) were purchased from Merck (Darmstadt, Germany) and Phenomenex (Torrance, CA, USA), respectively.

### 2.2 Field experiment in a greenhouse and sample collection

The field experiments were performed in compliance with Good Agriculture Practice (GAP) guidelines at two separate greenhouses sited approximately 64 km apart. The greenhouses were located in Buyeo (36°12′59″ N 126°48′10″ E; field I) and Taean (36°37′41″ N 126°18′39″ E; field II) in Chungcheongnamdo, Republic of Korea. The field experiments were conducted between February and March, with a sowing period of 5–8 months prior to experimentation. Each CAP and FBD trial consisted of three plots, each with an area of ≥10 m^2^ (each plot in field I: 9 m × 3 m; each plot in field II: 10.5 m × 1.4 m). The trials were separated by a buffer zone measuring at least 6 m^2^. Throughout the experiment, the temperature and humidity inside the greenhouse were monitored using CAS data loggers (EL-21CFR-2-LCD; Lascar Electronics, PA, USA). The average temperatures during the field experiment period were 10.5 ± 1.9 and 11.5 ± 3.6 °C in fields I and II, respectively. The humidity levels were 71.6 ± 4.6 % in field I and 74.7 ± 8.0 % in field II. Spray suspensions were prepared according to GAP guidelines and applied using an electric engine-dispensing sprayer (MSB20Li; Maruyama, Tokyo, Japan), as detailed in Table 1. Duplicate composite samples of the representative commodities for *A. scaber* were harvested from the treated plots at various days after final treatment (DATs): 0 (2 h), 1, 2, 3, 5, 7, and 10 days. Samples comprised at least 50 plants that were randomly selected across the plot, excluding the edges, with a minimum weight of 1 kg. All samples were placed in polyethylene bags in an icebox and transported to the laboratory. At the time of sample harvest, 50 bundles of each sample were weighed to assess their growth status. The average weight over a 10-day period was 49.7 ± 7.0 g in field I and 102.4 ± 11.2 g in field II (Fig. S1). The samples were homogenized with dry ice using a blender (Blixer®2; Lobot Coupe, Montceau-les-Mines, France) and then stored at −20 °C for approximately five months prior to analysis.

**Table 1:** GAP guidelines and MRLs of CAP and FBD in *A. scaber*.

Pesticide	Formulation	Application				PHI (days)	MRL (mg/kg)
		Dilution	Spray No.	Spray volume (L/m^2^)	Rate (g a.i./m^2^)		
Chlorantraniliprole	WG, 5 %	2000	1	0.4	0.01	7	7
Flubendiamide	SC, 20 %	2000	1	0.4	0.04	7	20

WG: water-dispersible granule; SC: suspension concentrate; a.i.: active ingredient; PHI: preharvest interval; MRL: maximum residue limit.

### 2.3 Washing and drying processes

The efficiency of residual pesticide removal was investigated using three washing procedures: running tap water, tap water bath, and neutral detergent. The samples were collected at 7 DATs, considering the pre-harvest intervals (PHIs) of both pesticides, as shown in Table 1 [20]. The running tap water procedure involved washing with water (pH 7.3) at a rate of 0.1 L/s for 1 min. For the tap water bath procedure, the samples were immersed and rinsed in 6 L of tap water (pH 7.3) for 1 min. In the neutral detergent washing procedure, the samples were dipped (immersed) in a 0.15 % neutral detergent solution for 1 min, followed by two rounds of washing (rinsing) with running tap water (pH 7.3) at 0.1 L/s for 30 s each. Subsequently, all the samples were air-dried for approximately 30 min at room temperature. The effect of the drying process on pesticide residues was investigated. The samples at 7 DATs were dried in a drying oven (LDO-250F; Daihan Labtech Co., Ltd., Namyangju, Republic of Korea) at 80 °C for 24 h without undergoing any washing process [20].

### 2.4 Residue analytical method

#### 2.4.1 Samples extraction and partitioning

Homogenized (20 g) or dried (5 g) samples were weighed and placed in a 500 mL centrifuge bottle. The dried sample was soaked in 10 mL of distilled water for 30 min to ensure thorough saturation. To extract CAP and FBD, 100 mL of ACN and 100 mL of ACE were added. The mixture was then vigorously shaken at 12,000 rpm for 3 min using a high-speed shaker (2010 Geno/Grinder®; SPEX SamplePrep, Mutton, NJ, USA). The homogenate was filtered under suction through filter paper, and the resulting filter cake was subsequently rinsed with 50 mL of ACN for CAP and 50 mL of ACE for FBD. The filtrate containing CAP was evaporated under vacuum at 40 °C, and the resulting concentrate was transferred to a separatory funnel, to which 50 mL of distilled water and 50 mL of saturated sodium chloride solution were added. The filtrate containing the FBD was diluted with 450 mL of distilled water and 50 mL of saturated sodium chloride solution. Each sample solution was subjected to sequential liquid−liquid partitioning with DCM. For CAP, partitioning was performed with 100 mL DCM, followed by additional partitioning with 50 mL DCM. For FBD, partitioning was performed twice, with 70 mL of DCM used each time. The DCM layers were dehydrated over anhydrous sodium sulfate and combined. The DCM fraction containing CAP and FBD was evaporated to dryness and subsequently dissolved in a 10 mL mixture of ETAC/HX (20/80, v/v) and a 10 mL mixture of ACE/HX (10:90, v/v), respectively, for further cleanup.

#### 2.4.2 Purification

To clean the CAP extract, a silica SPE cartridge (1 g, 6 cc) pre-conditioned with 10 mL of HX was employed. After adding 5 mL of the extract to the SPE cartridge, the residues were washed with a 15 mL mixture of ETAC/HX (30:70, v/v) and then eluted with a 10 mL mixture of ETAC/HX (40:60, v/v). For FBD, the dissolved extract was transferred to a glass column (11 mm i.d. × 40 cm) containing 5 g activated Florisil and 2 g anhydrous sodium sulfate. The residues were washed with a 50 mL mixture of ACE/HX (15:85, v/v), followed by elution using a 50 mL mixture of ACE/HX (25:75, v/v). Each eluate was concentrated to dryness at 40 °C under vacuum and then reconstituted with 10 mL and 20 mL of ACN for CAP and FBD, respectively. A 10 μL aliquot of the final solution was injected into a HPLC system equipped with a diode-array detector (HPLC-DAD), as shown in Table S1.

### 2.5 Method validation for quality assurance

The analytical methods used in this study were confirmed according to the guidelines of European SANTE/11312/2021 [21]. The method limit of quantitation (MLOQ) was determined based on the instrumental limit of quantitation (ILOQ; ng), injection volume (μL), sample mass (g), and final solution volume (mL) [22]. ILOQ was defined as the concentration at which the signal-to-noise ratio of pesticide intensity reached 10. Stock solutions with a concentration of 100 mg/L were prepared by dissolving 10.080 mg CAP (99.2 %) and 10.173 mg FBD (98.3 %) in 100 mL of ACN. Working solutions were prepared via the stepwise dilution of the stock solutions using ACN. The linearity of the calibration curve was assessed using the coefficient of determination (R^2^) obtained through linear regression in the concentration range of 0.05–10 mg/L for CAP and 0.25–20 mg/L for FBD. Recovery and repeatability experiments were conducted at two levels, 10MLOQ and MRL, with three replicates at each level. The storage stability of CAP and FBD in *A. scaber* was evaluated over approximately five months by analyzing samples fortified at 10MLOQ in triplicate.

### 2.6 Dissipation and biological half-lives for the pesticides in *A. scaber*

If the degradation of the pesticide agreed with pseudo-first-order kinetics [[23], [24], [25]], the dissipation constant (λ) of the pesticide in *A. scaber* was determined using the following equation:

(1) $$C_{t}=C_{0}\times \exp\left(-\lambda t\right)$$

where C_t_ is the concentration of the pesticide at time t (day), C_0_ is the initial concentration at 0 DATs, and λ is the dissipation constant (day^−1^). The mean and 95 % confidence interval (95 % CI) of the dissipation constant (λ) were calculated using regression analysis after evaluating the time-dependent residues of pesticides using the F-test and regression coefficient (R^2^).

The biological half-life (DT_50_) was calculated using the following equation:

(2) $${\text{DT}}_{50}=\ln\;2/\;\lambda =0.693\;/\;\lambda$$

### 2.7 Determination of PHRLs for the pesticides in *A. scaber*

The PHRLs of the pesticides were estimated based on the 95 % upper confidence limit (95 % UCL) of the dissipation constant (λ) and MRLs in *A. scaber* using the following equation [24,26]:

(3) $${\text{PHRL}}_{t}=\text{MRL}\;/\exp\left(\lambda_{\text{UCL}}\times \;t\right)$$

where the MRL in *A. scaber* is 7 mg/kg for CAP and 20 mg/kg for FBD [27].

### 2.8 Effect of processing methods on residual reduction

The efficiency of the pesticide residue removal during washing was determined using the following equation:

(4) Removalratebywashing(%)=(1−residueafterwashing/residuebeforewashing)×100

The processing factor (PF) in drying was calculated using the following equation:

(5) PF=residueafterdrying(mg/kg)/residuebeforedrying(mg/kg)

A PF value > 1 indicates the dominance of the concentration effect of the process, whereas a PF value < 1 signifies the reduction effect of the process [16,17].

The weight reduction ratio (WRR) was calculated to assess the extent of weight loss in the sample during the drying process. The compensated residue level (CRL) represents the residue in a fresh sample adjusted for weight loss during drying. The CRL was calculated by dividing the residue in the dried sample by the WRR value [20].

(6) WRR=weight before drying(g)/weight after drying(g)

WRR = weight before drying (g) / weight after drying (g) (6)

(7) CRL(mg/kg)=residueafterdrying(mg/kg)/WRR

The pesticide residue removal rate during drying was determined using the following equation:

(8) Removalrate(%)=(1−CRL/residuebeforedrying)×100

### 2.9 Statistics analysis

Statistical analysis was based on three independent experiments, and the results are presented as mean ± standard deviation (SD). One-way ANOVA and Duncan's multiple range tests were conducted to determine the significance of the differences (*p* < 0.05). Statistical analysis was performed using SPSS Statistics (version 18.0; SPSS Inc., Arming, NY, USA).

### 2.10 Estimation of the exposure and risk to pesticides by intake

Risk assessments were conducted by comparing long-term dietary exposure to pesticides with the relevant risk value. Thus, the chronic hazard quotient (HQ) was calculated using the following equation [16,23]:

(9) EDI=STMR×F/BW

(10) TMDI=∑MRL×F/BW

(11) HQ=EDIorTMDI/ADI×100

where EDI is the estimated daily intake of each agent (μg/kg b.w./day), STMR is the supervised trial median residue in this study (μg/g), F is the daily intake of a certain food type (g/day), BW is the body weight (kg b.w.), TMDI is the theoretical maximum daily intake (μg/kg b.w./day), MRL is the maximum residue level set by Korea (mg/kg or μg/g [27]), HQ is the chronic hazard quotient (%), and ADI is the acceptable daily intake (μg/kg b.w./day). The food intake and average body weight (62.5 kg b.w.) of Korean residents were obtained from the Korea Centers for Disease Control and Prevention [28]. ADIs are 2000 and 17 μg/kg b.w./day of CAP and FBD, respectively [6,7]. An HQ of <100 represents an acceptable risk level for human health [16].

## 3 Results and discussion

### 3.1 Validation of the residue analytical method

**Fig. 1:**
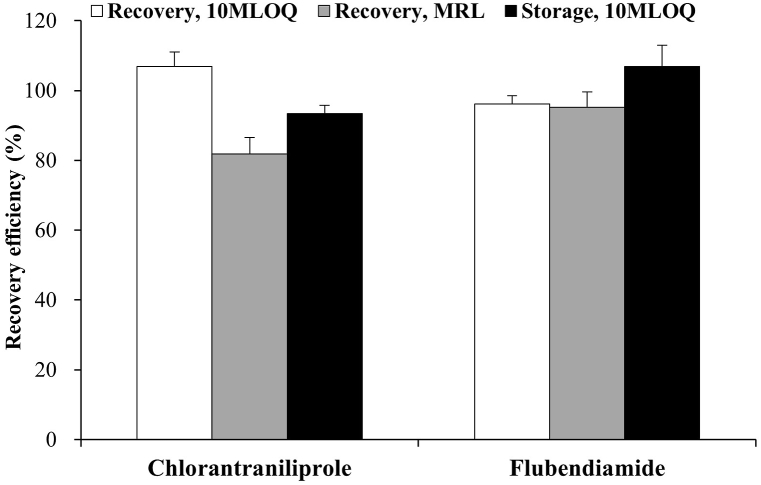
Recovery efficiency and storage stability for CAP and FBD in *A. scaber*. The fortification levels, 10MLOQ and MRL were 0.5 and 7 mg/kg for CAP, and 2.5 and 20 mg/kg for FBD, respectively (n = 3).

The residue analytical methods were validated using the following parameters: selectivity, MLOQ, calibration curve linearity, accuracy, precision, and storage stability. To assess selectivity, blank sample extracts were injected, and no interfering substances were detected at the retention time of the pesticides. The MLOQs were 0.05 and 0.25 mg/kg for CAP and FBD, respectively. The coefficients of determination (R^2^) for CAP and FBD were both 0.9999, indicating excellent linearity of the calibration curve, which satisfied the guideline criteria of >0.98. A recovery experiment was conducted to determine the accuracy and precision of the analytical method. The average recovery efficiencies were 81.9–106.9 % (coefficient of variation, CV ≤ 5.7 %) for CAP and 95.3–96.1 % (CV ≤ 4.6 %) for FBD, which were in good agreement with the average recovery values of 70–120 % and a coefficient of variation of ≤20 % specified in the guidelines (Fig. 1). The storage stability tests revealed that the recovery rate of CAP ranged from 91.5 % to 96.0 %, whereas that of FBD ranged from 99.8 % to 111.0 %. These results indicate that no decomposition of residues occurred in the samples during the storage periods of 142 and 111 days for CAP and FBD, respectively.

### 3.2 Residue characteristics and dissipation in *A. scaber*

The amount of pesticide deposited on plants is dependent on crop characteristics, such as plant surface area, structure, physiology, pesticide formulation type, pesticide use intensity by volume and concentration of spraying solution, spraying equipment/techniques, and droplet size [[29], [30], [31]]. The FBD residues at 0 DATs following foliar application were 3–10 times higher than those of CAP (Table 2). The concentration of the spray solution for FBD (20 % a.i., dilution factor 2,000) was 0.010 %, which was approximately three times higher than the concentration of 0.003 % for CAP (5 % a.i., dilution factor 2,000), as shown in Table 1. *A. scaber* is a leafy vegetable that undergoes multiple discrete harvests with non-harvest periods between them. The samples analyzed in this study were collected during the first and third harvest seasons in fields I and II, respectively. Leaves harvested in field II exhibited higher weights and greater hardness and roughness compared to those harvested in field I. The higher residue levels observed in field I may be attributed to the higher foliar deposit rate of the spray solution, which results from the lower weight and smoother, softer surfaces of the individual samples compared with those in field II. Therefore, the differences in the initial residues were probably due to differences in the concentration of the spray solution and foliar deposition patterns influenced by leaf surface characteristics and size.

**Table 2:** Residues of CAP and FBD in *A. scaber*.

Days after final treatment (DAT)	Residue levels (mg/kg, mean ± standard deviation, n = 3)			
	CAP		FBD	
	Field I	Field II	Field I	Field II
0	4.7 ± 0.2	1.8 ± 0.3	49.9 ± 2.3	4.1 ± 0.8
1	3.9 ± 0.1	1.5 ± 0.1	39.8 ± 3.6	3.6 ± 0.5
2	3.5 ± 0.1	1.3 ± 0.3	32.2 ± 1.5	3.2 ± 0.3
3	3.3 ± 0.4	1.3 ± 0.2	29.7 ± 1.4	2.8 ± 0.3
5	2.8 ± 0.3	1.1 ± 0.2	25.8 ± 1.5	2.4 ± 0.4
7	2.5 ± 0.5	0.7 ± 0.1	18.9 ± 1.3	2.4 ± 0.3
10	1.9 ± 0.2	0.6 ± 0.1	17.7 ± 1.8	2.0 ± 0.5
λ±95%CI	−0.0831 ± 0.0148	−0.1151 ± 0.0208	−0.1014 ± 0.0330	−0.0700 ± 0.0240

λ: dissipation constant (day^−1^); 95%CI: 95 % confidence interval.

The residues in *A. scaber* gradually decreased the day after foliar application. The dissipation proportions of CAP and FBD over 10 days were 60–68 % and 53–64 %, respectively. Similarly, the dissipation proportions of acrinathrin and metaflumizone in *A. scaber* were reported to be 56–83 % [24]. According to the GAP guidelines, the recommended PHI is 7 days for both CAP and FBD. The residue levels at 7 DATs for both pesticides were below the MRLs of 7 and 20 mg/kg for CAP and FBD, respectively.

**Fig. 2:**
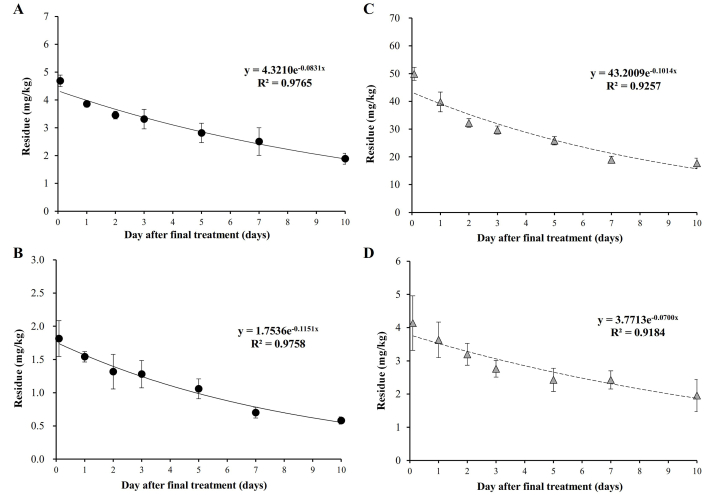
Time-dependent residual patterns of CAP (A, Field I; B, Field II) and FBD (C, Field I; D, Field II) in *A. scaber*.

As shown in Fig. 2, the dissipation of CAP and FBD followed pseudo-first-order kinetics, with R^2^ values ranging from 0.9184 to 0.9765. The DT_50_ values were 8.3 (95 % CI, 7.1–10.2) and 6.0 (95 % CI, 5.1–7.4) days for CAP in fields I and II, respectively, and 6.8 (95 % CI, 5.2–10.1) and 9.9 (95 % CI, 7.4–15.1) days for FBD. CAP exhibits long half-lives in perilla leaves (19.3 days [10]) because of its systemic properties [32]. However, it rapidly degrades in fruits, such as strawberry (4.7–6.4 days [33]) and tomato (3.3 days [34]). In contrast to other diamines, FBD is not fully systemic in plants [32]. It tends to rapidly dissipate in several plants, including kale (2.4–2.7 days [11]) and tomato (3.9 days [13]). Pesticide dissipation in plants is attributed to phase partitioning, intermedia transport, chemical degradation, biotransformation, and dilution by growth [35]. Growth dilution has the most impact, particularly in rapidly growing crops, such as lettuce and spinach, thus resulting in relatively short half-lives. For example, the half-life of CAP in spinach was 2.6–4.0 days [23]. Although *A. scaber* samples in field II were approximately twice as heavy as those in field I, the samples collected from fields I and II exhibited deviations of 14 % and 11 %, respectively, over the 10-day period. In this study, negligible variation in the sample weight over time suggests that the dilution effect on pesticide residues can be disregarded. Therefore, the slower dissipation of pesticide residues in *A. scaber* than in other crops may be attributed to its lower enzymatic degradation capacity, resulting in prolonged persistence. Previous research has reported longer half-lives in *A. scaber*, such as 3.8–9.2, 4.5–5.9, 8.5, 5.8, and 7.6 days for acrinathrin, metaflumizone, methoxyfenzide, novaluron, and pymetrozine, respectively, which were determined by excluding any potential dilution effect [24,36,37]. These results are consistent with those of the present study.

### 3.3 Establishment of PHRLs in *A. scaber*

The average residue levels at 7 DATs in both fields were 1.6 mg/kg for CAP and 10.7 mg/kg for FBD, which were 23 % and 53 % of their MRLs, respectively. Despite the difference in initial residues, no significant difference was observed between the dissipation constants of the two fields. According to the guidelines of the Ministry of Food and Drug Safety in Korea, when the average residue at the PHI is between 20 % and 60 % of the MRL, the PHRLs are determined using the 95 % UCL of the dissipation constant, which is calculated from the average daily residue in both fields [26,38]. In this regard, the dissipation constants based on the residues in both fields were −0.0920 (95 % CI, −0.1049 to −0.0791) and −0.1010 (95 % CI, −0.1297 to −0.0723) for CAP and FBD, respectively. The PHRLs for *A. scaber* 7 days before harvest were 12.2 and 33.2 mg/kg for CAP and FBD, respectively (Table S2). The PHRLs 7 days before harvest were approximately 1.74 times higher than the MRLs for CAP and 1.66 times higher for FBD. Hence, PHRLs can be extrapolated from the MRLs of different countries and regulations based on these ratios. The results obtained in this study can serve as a valuable guideline for producing safe agricultural commodities before harvesting and will facilitate the enforcement of measures to prevent agricultural commodities from exceeding the MRL before distribution, thereby ensuring consumer food safety.

### 3.4 Removal efficiency of residues by washing

Washing is a pre-treatment in any type of processing and is used to easily remove pesticide residues from agricultural products in households. Various washing methods, including running, dipping, rubbing, and ultra-sonication, with different agents such as tap water, chlorinated water, ozonated water, alcohol, acetic acid, ascorbic acid, citric acid, detergent, hydrogen peroxide, sodium chloride, and sodium carbonate, have been proposed for the removal of residual pesticides [17,18].

**Fig. 3:**
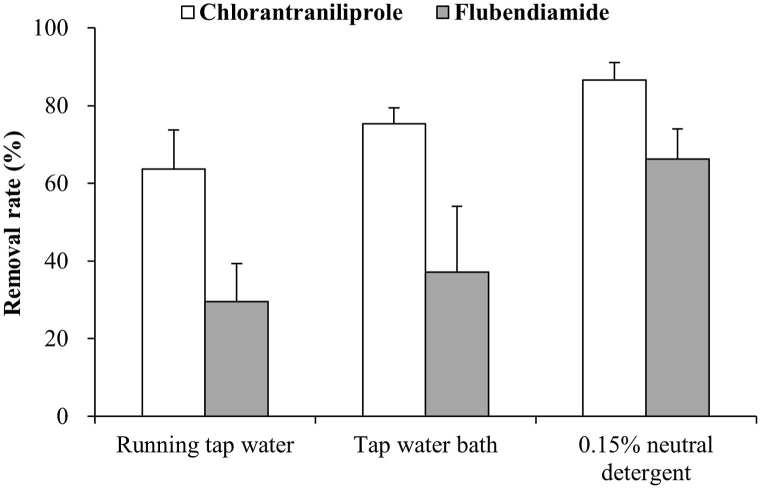
Removal efficiency of CAP and FBD in *A. scaber* across fields using different household washing processes.

Processing studies should simulate industrial or domestic practices as closely as possible [39]. Therefore, we investigated the removal efficiencies of pesticide residues using three washing methods commonly used at home: running under tap water and dipping in tap water or a neutral detergent. As shown in Fig. 3, running under tap water, a tap water bath, and using neutral detergent washing removed 63.7 %, 75.5 %, and 86.5 % of CAP, respectively. Washing with a 0.15 % neutral detergent solution was the most effective method for CAP removal from *A. scaber*. Furthermore, washing with a neutral detergent resulted in the highest FBD removal efficiency (66.2 %). Detergents help to emulsify fat-soluble pesticides deposited on leaf surfaces, reduce surface tension, and facilitate washing. However, the neutral detergent washing procedure includes an additional step of rinsing the detergent with running tap water. Therefore, the removal efficiencies achieved with the neutral detergent alone were estimated to be 58.6 % and 51.4 % for CAP and FBD, respectively. Consequently, this study demonstrated that the most effective single-step approach for eliminating residues in *A. scaber* was tap water bath washing for CAP and neutral detergent washing for FBD. Yang et al. [19] reported that washing leafy vegetables under running water resulted in the highest CAP residue removal rate. These results may be because most pesticides tend to remain on the surface of the plant rather than penetrate plant tissues, particularly when leafy vegetables are contaminated by soaking in a pesticide solution. The gentle flow of running water can effectively remove loosely attached residues [20,40,41]. Although the variations in efficacy among studies, including the findings of the present study, can be attributed to the nuances of the washing procedures, a consistent trend across several studies suggests the potential to achieve adequate residue removal through water-based washing alone. The effectiveness of washing depends on the physico-chemical properties (i.e., water solubility and polarity) of the active substances, their method of penetration, biological properties of crops, and conditions of the technological processes [17]. The removal rates varied between 63.7 % and 86.5 % for CAP and between 29.5 % and 66.2 % for FBD across the different washing processes. The higher removal rates of CAP can be attributed to its relatively higher water solubility (1.0 mg/L) and greater polarity (logP 2.76) compared to FBD (water solubility, 0.03 mg/L; logP 4.2), even though both pesticides can penetrate into plant tissues [42].

### 3.5 Removal rate of residues through the drying process

After drying, both pesticide residues exhibited an increase, yielding PF values of 2.6 ± 0.6 and 2.0 ± 0.1 for CAP and FBD in field I, and 5.1 ± 1.0 and 5.7 ± 1.6 in field II, respectively, with no significant difference between pesticides (Table 3). The difference in PF values between the two fields was attributed to the variance in water content of the fresh samples. Particularly, the samples from field I had a water content of approximately 82 %, whereas the samples from field II had a water content of approximately 92 %, as indicated by the WRR values. The alteration of the residue levels via thermal processing is influenced by the physico-chemical properties of the pesticide (volatility, boiling point, and solubility), process conditions (temperature, duration, and presence of fluids), and system specificity [17]. Both pesticides exhibited low water solubility (1.0 mg/L for CAP; 0.03 mg/L for FBD) and poor volatility (vapor pressure 2.1 × 10^−8^ mPa for CAP; 3.8 × 10^−7^ mPa for FBD) [42]. Therefore, the results demonstrated an increase in the residue attributed to water loss during drying, rather than a decrease due to thermal decomposition and residue evaporation.

**Table 3:** Removal efficiency (n = 3) from *A. scaber* using washing and drying in an oven.

Washing		CAP		FBD	
		Field I	Field II	Field I	Field II
Residue (mg/kg)	before processing	2.5 ± 0.5	0.7 ± 0.1	18.9 ± 1.3	2.4 ± 0.3
	Running tap water	0.9 ± 0.1	0.2 ± 0.1	13.5 ± 2.4	1.7 ± 0.1
	Tap water bath	0.5 ± 0.03	0.2 ± 0.02	14.6 ± 0.2	1.2 ± 0.04
	0.15 % neutral detergent	0.3 ± 0.1	0.1 ± 0.05	7.7 ± 0.6	0.6 ± 0.01
	after drying	6.2 ± 0.4	3.6 ± 1.0	38.0 ± 0.6	13.5 ± 2.5
PF		2.6 ± 0.6	5.1 ± 1.0	2.0 ± 0.1	5.7 ± 1.6
WRR		4.8 ± 0.9	10.7 ± 0.7	3.9 ± 2.0	11.2 ± 3.0
CRL (mg/kg)		1.3 ± 0.1	0.3 ± 0.1	9.7 ± 0.2	1.2 ± 0.2
Removal rate (%)		46.4 ± 11.9	52.3 ± 9.3	48.4 ± 3.7	49.2 ± 14.5

PF: processing factor; WRR: weight reduction ratio; CRL: compensated residue level.

The removal rates, excluding the concentration factor owing to water loss, were determined by comparing the residue before drying with the CRL. CRLs (mg/kg) were determined by adjusting the residues in the dried samples using WRR. The removal efficiencies achieved by thermal decomposition and evaporation during drying were 46.4–52.3 % and 48.4–49.2 % for CAP and FBD, respectively, across both fields (Table 3).

In summary, the drying process resulted in an approximately 2–6-fold increase in the CAP and FBD concentrations in *A. scaber*. This was primarily due to an approximately 4–11-fold concentration increase resulting from water loss, which was partially countered by approximately 50 % dissipation due to thermal decomposition and evaporation.

### 3.6 Dietary exposure and risk assessment

The chronic health risks from dietary pesticide exposure for the general population were assessed by determining the EDI, based on residue levels in *A. scaber*, and the TMDI using the established MRLs, owing to the unavailability of supervised trial median residue data for the target pesticides in registered crops in Korea. Acute health risks were not evaluated in this study because there is no established acute reference dose for CAP due to its low acute toxicity.

The EDIs were calculated using STMRs of 1.4 and 10.1 mg/kg for CAP and FBD, respectively, with a PHI of 7 days according to GAP guidelines. As shown in Table 4, the EDIs for CAP and FBD across all age groups were 0.0188 and 0.1341 μg/kg b.w./day, which accounted for 0.001 % and 0.79 % of HQs, respectively. Among the different age groups, the highest EDIs were observed in the age ranges of 1–2 and 50–64 years, where the intake per body weight was approximately five times higher than in the other age groups. These results indicate that the residues of both pesticides in *A. scaber* have minimal health impacts on the general population. The TMDIs of CAP and FBD were 11.7672 and 7.5179 μg/kg b.w./day, respectively, which corresponds to 0.07 % and 44.22 % of HQs, which are <100 %. Despite its low TMDI, the high HQ of FBD was attributed to its high chronic toxicity. The risk associated with *A. scaber* contributed 3.53 % and 6.67 % of the total risk for CAP and FBD, respectively (Tables S3 and S4). Overall, the health effects of both pesticides on the general population were within acceptable risk levels.

**Table 4:** Chronic hazard quotients of CAP and FBD with the consumption of *A. scaber* in Korea.

Ages	Food daily intake (g/day)	Body weight (kg b.w.)	EDI (μg/kg b.w./day)		HQ_EDI_ (%)	
			CAP	FBD	CAP	FBD
1–2	0.38	12.2	0.0441	0.3146	0.0022	1.85
3–5	0.04	18.5	0.0031	0.0218	0.0002	0.13
6–11	0.25	34.1	0.0104	0.0741	0.0005	0.44
12–18	0.05	60.6	0.0012	0.0083	0.0001	0.05
19–29	0.28	68.6	0.0058	0.0412	0.0003	0.24
30–49	0.42	69.2	0.0086	0.0613	0.0004	0.36
50–64	1.92	65.6	0.0414	0.2957	0.0021	1.74
≥65	1.12	60.2	0.0263	0.1879	0.0013	1.11
All	0.83	62.5	0.0188	0.1341	0.0009	0.79

EDI: estimated daily intake; TMDI: theoretical maximum daily intake; HQ: chronic hazard quotient.

## 4 Conclusion

In this study, the residue characteristics, processing and risk assessment of CAP and FBD were investigated in *A. scaber*. Analytical methods, including extraction, partitioning, purification, and instrumental analysis using HPLC-DAD, have been developed and validated for the determination of residues in *A. scaber*. The half-lives of CAP and FBD in *A. scaber* were 6.0–8.3 and 6.8–9.9 days, respectively, which are longer than other crops due to their prolonged persistence and lower enzymatic degradation capacity. The residues at the PHI (7 DATs), as recommended by the GAP guidelines, were below the MRLs (CAP, ≤7 mg/kg; FBD, ≤20 mg/kg) in Korea. The PHRLs for *A. scaber* 7 days before harvest were 12.2 and 33.2 mg/kg for CAP and FBD, respectively. Washing with a neutral detergent solution was the most effective method for removing CAP and FBD from *A. scaber*; however, water-based washing also showed satisfactory removal effects. The PFs in the drying process ranged from 2 to 6 for both pesticides, which was attributed to a 4–11-fold increase in concentration owing to water loss and a 46–52 % decrease owing to thermal decomposition and evaporation. Therefore, if *A. scaber* were washed with a neutral detergent followed by drying, 93.2 % and 82.7 % of the CAP and FBD, respectively, could be removed, resulting in negligible residues. Dietary exposure to CAP and FBD through the consumption of *A. scaber* or several agricultural commodities with MRLs set in Korea was below the relevant risk value, ADI (i.e., <100 % of HQs), indicating the least possibility of risk for the general population in Korea. The results of this study could be applied to the cultivation of safe agricultural commodities and to ensure pesticide safety in food.

## Data availability statement

The data included in article and supplementary material in article.

## Funding

This study was supported by Wonkwang University in 2024.

## CRediT authorship contribution statement

**Seung-Hyun Yang:** Writing – original draft, Validation, Methodology, Formal analysis, Data curation, Conceptualization. **Hoon Choi:** Writing – review & editing, Supervision, Resources, Project administration, Methodology, Funding acquisition, Data curation, Conceptualization.

## Declaration of competing interest

The authors declare that they have no known competing financial interests or personal relationships that could have appeared to influence the work reported in this paper.
