# Supplementary material for: Insecticides chlorantraniliprole and flubendiamide in Aster scaber: Dissipation kinetics, processing effects, and risk assessment
Source: Heliyon. 2024 Jun 17;10(12):e33216. doi: 10.1016/j.heliyon.2024.e33216 (PMC11252733; doi:10.1016/j.heliyon.2024.e33216)
Supplement: Multimedia component 1 [file mmc1.docx]

**Supplemental Material**

Table S1. Instrumental conditions for residue analysis in *A. scaber*.

| Pesticide | Chlorantraniliprole | Flubendiamide |
| --- | --- | --- |
| Instrument | Agilent 1100 series (Atlanta, GA, USA) | |
| Column | Brownlee SPP C18  (100 × 4.6 mm, 2.7 μm; PerkinElmer, Waltham, MA, USA) | Gemini-NX C18  (150 × 4.6 mm, 3 μm; Phenomenex, Torrance, CA, USA) |
| Column temp. | 25 ℃ | 25 ℃ |
| Mobile phase | ACN/Water (40:60, v/v) | ACN/Water (65:35, v/v) |
| Flow rate | 0.8 mL/min | 0.6 mL/min |
| Injection volume | 10 µL | 10 µL |
| Wavelength | 220 nm | 253 nm |
| Retention time | 8.4 min | 8.8 min |

Table S2. Pre-harvest residue limits for CAP and FBD in *A. scaber*.

| Pesticide | Pre-harvest residue limit (mg/kg) | | | | MRLs (mg/kg) | λ_UCL_ |
| --- | --- | --- | --- | --- | --- | --- |
|  | 10 days  before harvest | 7 days  before harvest | 5 days  before harvest | 3 days  before harvest |  |  |
| CAP | 15.4 | 12.2 | 10.4 | 8.9 | 7 | −0.0791 |
| FBD | 41.2 | 33.2 | 28.7 | 24.8 | 20 | −0.0723 |

MRL: maximum residue limit; λ_UCL_: 95% UCL of the dissipation constant.

Table S3. Risk and exposure assessment of CAP using TMDI in Korea.

| Agricultural commodity | Food daily intake (g/day) | MRL (mg/kg) | TMDI (μg/kg b.w./day) | HQ_TMDI_ (%) | Agricultural commodity | Food daily intake (g/day) | MRL (mg/kg) | TMDI (μg/kg b.w./day) | HQ_TMDI_ (%) |
| --- | --- | --- | --- | --- | --- | --- | --- | --- | --- |
| Amaranth leaves | 0.13 | 10 | 0.0208 | 0.001 | Lemon | 0.08 | 1 | 0.0013 | 0.0001 |
| Apple | 33.91 | 2 | 1.0851 | 0.05 | Lettuce, head | 1.73 | 4 | 0.1107 | 0.006 |
| Apricot | 0.2 | 0.7 | 0.0022 | 0.0001 | Lettuce, leaves | 7.19 | 7 | 0.8053 | 0.04 |
| Aronia | 0.11 | 1 | 0.0018 | 0.0001 | Mandarin | 11.47 | 1 | 0.1835 | 0.01 |
| Asparagus | 0.01 | 0.3 | 0 | 0.000002 | Mango | 0.34 | 0.2 | 0.0011 | 0.0001 |
| Avocado | 0.37 | 0.3 | 0.0018 | 0.0001 | Marsh mallow | 0.31 | 10 | 0.0496 | 0.002 |
| Balsam pear | 0.02 | 0.2 | 0.0001 | 0.000003 | Melon | 2.07 | 0.2 | 0.0066 | 0.0003 |
| Barley | 5.71 | 0.05 | 0.0046 | 0.0002 | Millet | 0.54 | 0.05 | 0.0004 | 0.00002 |
| Beans | 34.04 | 0.3 | 0.1634 | 0.008 | Mulberry | 0.06 | 1 | 0.001 | 0 |
| Beet, leaves | 0.02 | 7 | 0.0022 | 0.0001 | Nuts | 5.72 | 0.02 | 0.0018 | 0.0001 |
| Beet, roots | 0.85 | 0.2 | 0.0027 | 0.0001 | Onion | 31.75 | 0.05 | 0.0254 | 0.001 |
| Blueberry | 0.71 | 1 | 0.0114 | 0.0006 | Oyster mushroom | 1.12 | 0.2 | 0.0036 | 0.0002 |
| Bonnet bellflower | 0.15 | 0.15 | 0.0004 | 0.00002 | Passion fruit | 0.1 | 2 | 0.0032 | 0.0002 |
| Broccoli | 0.79 | 3 | 0.0379 | 0.002 | Peanut | 0.59 | 0.06 | 0.0006 | 0.00003 |
| Buckwheat | 0.02 | 3 | 0.001 | 0 | Perilla leaves | 3.06 | 10 | 0.4896 | 0.02 |
| Burdock, roots | 0.7 | 0.05 | 0.0006 | 0.00003 | Perilla seed | 0.25 | 0.2 | 0.0008 | 0.00004 |
| Cabbage, head | 8.66 | 1.5 | 0.2078 | 0.01 | Perillae folium | 0.01 | 10 | 0.0016 | 0.0001 |
| Carrot | 8.51 | 0.05 | 0.0068 | 0.0003 | Plum | 3.17 | 0.5 | 0.0254 | 0.001 |
| Celery | 0.06 | 7 | 0.0067 | 0.0003 | Pome fruits | 131.3 | 1 | 2.1008 | 0.11 |
| Chamnamul | 0.16 | 10 | 0.0256 | 0.001 | Pomegranate | 0.04 | 0.5 | 0.0003 | 0.00002 |
| Chard | 0.13 | 5 | 0.0104 | 0.0005 | Potato | 22.18 | 0.05 | 0.0177 | 0.001 |
| Cherry | 0.96 | 0.5 | 0.0077 | 0.0004 | Proso millet | 0.12 | 0.3 | 0.0006 | 0.00003 |
| Chicory | 0.11 | 10 | 0.0176 | 0.001 | Pumpkin young leaves | 0.23 | 15 | 0.0552 | 0.003 |
| Chinese magnolia vine | 0.01 | 0.5 | 0.0001 | 0.000004 | Radish, leaves | 1.46 | 10 | 0.2336 | 0.01 |
| *A. scaber* | 0.83 | 7 | 0.093 | 0.005 | Radish, roots | 20.8 | 0.05 | 0.0166 | 0.0008 |
| Citrus fruits | 2.97 | 0.6 | 0.0285 | 0.001 | Rape seed | 0.43 | 2 | 0.0138 | 0.0007 |
| Coffee bean | 0.79 | 0.03 | 0.0004 | 0.00002 | Red been | 0.31 | 0.05 | 0.0002 | 0.00001 |
| Corn | 6.11 | 0.05 | 0.0049 | 0.0002 | Rice | 121.88 | 0.5 | 0.975 | 0.05 |
| Cranberry | 0.13 | 0.7 | 0.0015 | 0.0001 | Sesam seed | 0.62 | 0.1 | 0.001 | 0 |
| Crown daisy | 0.44 | 4 | 0.0282 | 0.001 | Sorghum | 0.28 | 3 | 0.0134 | 0.0007 |
| Cucumber | 17.6 | 0.5 | 0.1408 | 0.007 | Soy bean | 2.75 | 0.05 | 0.0022 | 0.0001 |
| Dragon fruit | 0.02 | 0.7 | 0.0002 | 0.00001 | Spinach | 4.19 | 5 | 0.3352 | 0.02 |
| Eggplant | 2.6 | 0.2 | 0.0083 | 0.0004 | Squash | 9.58 | 0.7 | 0.1073 | 0.005 |
| Fig | 0.12 | 0.2 | 0.0004 | 0.00002 | Stalk and stem vegetables | 268.12 | 0.7 | 3.0029 | 0.15 |
| Garlic | 4.41 | 0.05 | 0.0035 | 0.0002 | Strawberry | 2.09 | 1 | 0.0334 | 0.002 |
| Ginger | 0.28 | 0.15 | 0.0007 | 0.00003 | Sunflower seed | 0.05 | 2 | 0.0016 | 0.0001 |
| Grapes | 7.16 | 2 | 0.2291 | 0.01 | Sweet pepper | 0.7 | 1 | 0.0112 | 0.0006 |
| Green & red pepper (fresh) | 4.62 | 1 | 0.0739 | 0.004 | Sweet potato | 11.54 | 0.05 | 0.0092 | 0.0005 |
| Green garlic | 0.04 | 0.05 | 0 | 0.000002 | Sweet potato stalk | 1.05 | 0.2 | 0.0034 | 0.0002 |
| Jujube | 0.36 | 0.7 | 0.004 | 0.0002 | Taro | 0.14 | 0.05 | 0.0001 | 0.00001 |
| Kale | 0.03 | 10 | 0.0048 | 0.0002 | Tomato | 14.6 | 1 | 0.2336 | 0.01 |
| Kimchi cabbage | 5.25 | 1 | 0.084 | 0.004 | Turnip | 0.01 | 0.07 | 0 | 0.000001 |
| Kiwifruit | 1.99 | 0.5 | 0.0159 | 0.0008 | Watermelon | 14.5 | 0.05 | 0.0116 | 0.0006 |
| Korean lemon | 0.55 | 1 | 0.0088 | 0.0004 | Welsh onion | 10.85 | 2 | 0.3472 | 0.02 |
| Korean melon | 10.23 | 1 | 0.1637 | 0.008 | Wild garlic leaves | 0.08 | 10 | 0.0128 | 0.0006 |
| Korean plum | 0.16 | 0.7 | 0.0018 | 0.0001 | Yam | 0.51 | 0.05 | 0.0004 | 0.00002 |
| Korean wasabi, leaves | 0.1 | 0.15 | 0.0002 | 0.00001 | **Sum** | **-** | **-** | **11.7672** | **0.07** |

MRL: maximum residue limit; TMDI: theoretical maximum daily intake; HQ: chronic hazard quotient.

Table S4. Risk and exposure assessment of FBD using TMDI in Korea.

| Agricultural commodity | Food daily intake (g/day) | MRL (mg/kg) | TMDI (μg/kg b.w./day) | HQ_TMDI_ (%) | Agricultural commodity | Food daily intake (g/day) | MRL (mg/kg) | TMDI (μg/kg b.w./day) | HQ_TMDI_ (%) |
| --- | --- | --- | --- | --- | --- | --- | --- | --- | --- |
| Apple | 33.91 | 1 | 0.5426 | 3.19 | Lettuce, leaves | 7.19 | 10 | 1.1504 | 6.77 |
| Broccoli | 0.79 | 3 | 0.0379 | 0.22 | Mandarin | 11.47 | 1 | 0.1835 | 1.08 |
| Butterbur | 0.29 | 15 | 0.0696 | 0.41 | Melon | 2.07 | 1 | 0.0331 | 0.19 |
| Cabbage, head | 8.66 | 0.3 | 0.0416 | 0.24 | Nuts | 5.72 | 0.1 | 0.0092 | 0.05 |
| Cherry | 0.96 | 2 | 0.0307 | 0.18 | Peach | 13 | 0.7 | 0.1456 | 0.86 |
| Chives | 2.82 | 3 | 0.1354 | 0.8 | Pear | 8.87 | 1 | 0.1419 | 0.83 |
| *A. scaber* | 0.83 | 20 | 0.2656 | 1.56 | Perilla leaves | 3.06 | 15 | 0.7344 | 4.32 |
| Corn | 6.11 | 0.05 | 0.0049 | 0.03 | Persimmon | 9.64 | 0.5 | 0.0771 | 0.45 |
| Cucumber | 17.6 | 1 | 0.2816 | 1.66 | Plum | 3.17 | 1 | 0.0507 | 0.3 |
| Grapes | 7.16 | 1 | 0.1146 | 0.67 | Radish, leaves | 1.46 | 7 | 0.1635 | 0.96 |
| Green & red pepper (fresh) | 4.62 | 1 | 0.0739 | 0.43 | Radish, roots | 20.8 | 0.05 | 0.0166 | 0.1 |
| Jujube | 0.36 | 2 | 0.0115 | 0.07 | Rice | 121.88 | 0.5 | 0.975 | 5.74 |
| Kales | 0.03 | 0.7 | 0.0003 | 0.002 | Soy beans | 2.75 | 0.1 | 0.0044 | 0.03 |
| Kimchi cabbage | 5.25 | 1 | 0.084 | 0.49 | Spinach | 4.19 | 10 | 0.6704 | 3.94 |
| Kiwifruit | 1.99 | 1 | 0.0318 | 0.19 | Strawberry | 2.09 | 1 | 0.0334 | 0.2 |
| Korean melon | 10.23 | 1 | 0.1637 | 0.96 | Sweet pepper | 0.7 | 1 | 0.0112 | 0.07 |
| Korean plum | 0.16 | 1 | 0.0026 | 0.02 | Tomato | 14.6 | 0.7 | 0.1635 | 0.96 |
| Korean wasabi, leaves | 0.1 | 20 | 0.032 | 0.19 | Watermelon | 14.5 | 1 | 0.232 | 1.36 |
| Lettuce, head | 1.73 | 10 | 0.2768 | 1.63 | Welsh onion | 10.85 | 3 | 0.5208 | 3.06 |
|  |  |  |  |  | **Sum** | **-** | **-** | **7.5159** | **44.22** |

MRL: maximum residue limit; TMDI: theoretical maximum daily intake; HQ: chronic hazard quotient.

**Figure Legends**


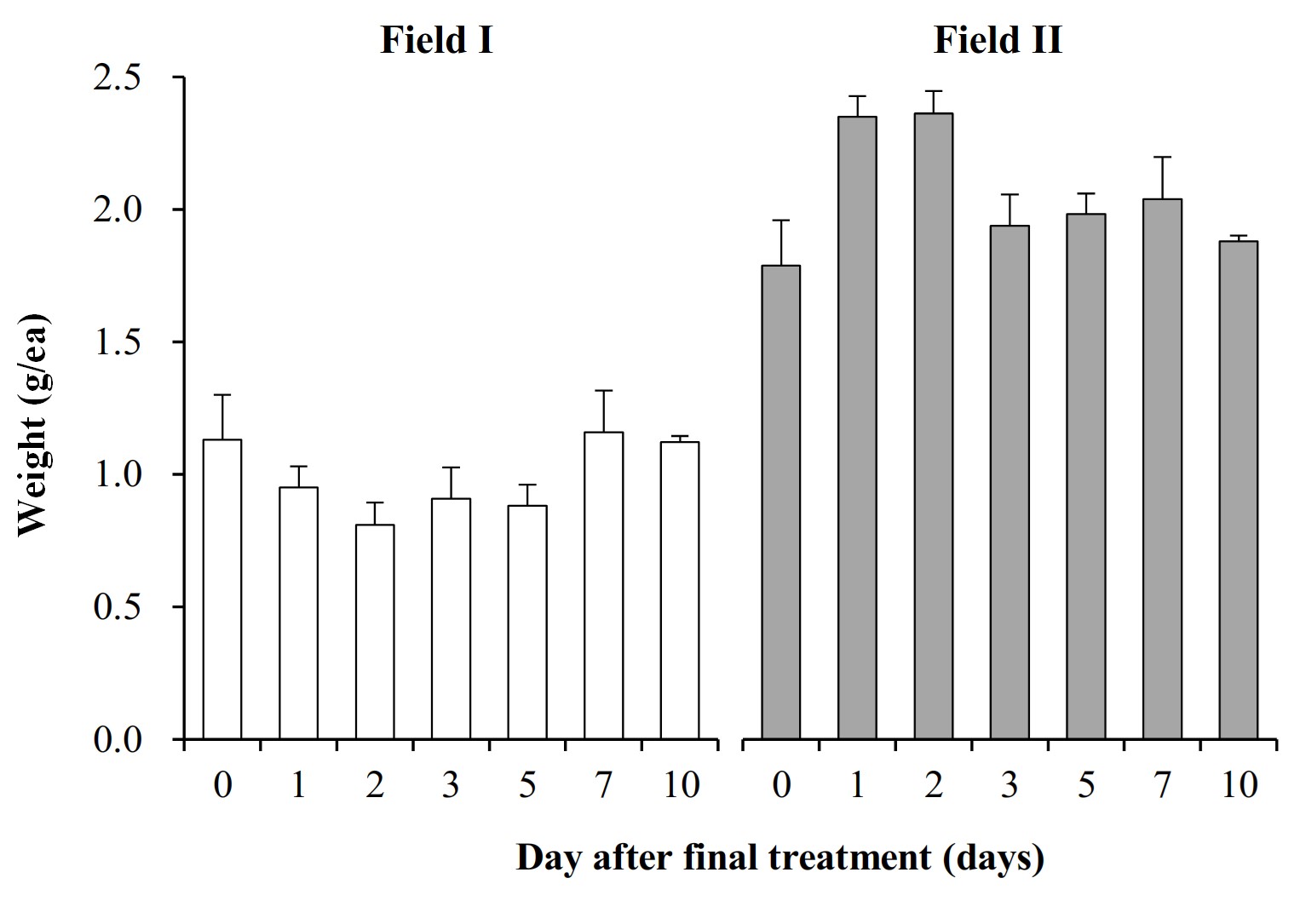


Fig. S1. Weight distribution of *A.* *scaber* during the experimental period.
